# A Machine Learning Explanation of the Pathogen-Immune Relationship of SARS-CoV-2 (COVID-19), and a Model to Predict Immunity and Therapeutic Opportunity: A Comparative Effectiveness Research Study

**DOI:** 10.2196/23582

**Published:** 2020-10-19

**Authors:** Eric Luellen

**Affiliations:** 1 Bioinformatix Interlochen, MI United States

**Keywords:** infectious disease, SARS-CoV-2, COVID-19, public health, immunity, mass vaccinations, therapeutics, stem-cell growth factor-beta

## Abstract

**Background:**

Approximately 80% of those infected with COVID-19 are immune. They are asymptomatic unknown carriers who can still infect those with whom they come into contact. Understanding what makes them immune could inform public health policies as to who needs to be protected and why, and possibly lead to a novel treatment for those who cannot, or will not, be vaccinated once a vaccine is available.

**Objective:**

The primary objectives of this study were to learn if machine learning could identify patterns in the pathogen-host immune relationship that differentiate or predict COVID-19 symptom immunity and, if so, which ones and at what levels. The secondary objective was to learn if machine learning could take such differentiators to build a model that could predict COVID-19 immunity with clinical accuracy. The tertiary purpose was to learn about the relevance of other immune factors.

**Methods:**

This was a comparative effectiveness research study on 53 common immunological factors using machine learning on clinical data from 74 similarly grouped Chinese COVID-19–positive patients, 37 of whom were symptomatic and 37 asymptomatic. The setting was a single-center primary care hospital in the Wanzhou District of China. Immunological factors were measured in patients who were diagnosed as SARS-CoV-2 positive by reverse transcriptase-polymerase chain reaction (RT-PCR) in the 14 days before observations were recorded. The median age of the 37 asymptomatic patients was 41 years (range 8-75 years); 22 were female, 15 were male. For comparison, 37 RT-PCR test–positive patients were selected and matched to the asymptomatic group by age, comorbidities, and sex. Machine learning models were trained and compared to understand the pathogen-immune relationship and predict who was immune to COVID-19 and why, using the statistical programming language R.

**Results:**

When stem cell growth factor-beta (SCGF-β) was included in the machine learning analysis, a decision tree and extreme gradient boosting algorithms classified and predicted COVID-19 symptom immunity with 100% accuracy. When SCGF-β was excluded, a random-forest algorithm classified and predicted asymptomatic and symptomatic cases of COVID-19 with 94.8% AUROC (area under the receiver operating characteristic) curve accuracy (95% CI 90.17%-100%). In total, 34 common immune factors have statistically significant associations with COVID-19 symptoms (all c<.05), and 19 immune factors appear to have no statistically significant association.

**Conclusions:**

The primary outcome was that asymptomatic patients with COVID-19 could be identified by three distinct immunological factors and levels: SCGF-β (>127,637), interleukin-16 (IL-16) (>45), and macrophage colony-stimulating factor (M-CSF) (>57). The secondary study outcome was the suggestion that stem-cell therapy with SCGF-β may be a novel treatment for COVID-19. Individuals with an SCGF-β level >127,637, or an IL-16 level >45 and an M-CSF level >57, appear to be predictively immune to COVID-19 100% and 94.8% (AUROC) of the time, respectively. Testing levels of these three immunological factors may be a valuable tool at the point of care for managing and preventing outbreaks. Further, stem-cell therapy via SCGF-β and M-CSF appear to be promising novel therapeutics for patients with COVID-19.

## Introduction

Asymptomatic patients who are infected with SARS-CoV-2 have neither clinical symptoms nor abnormal chest imaging. However, these patients have the same infectivity as infected patients with symptoms [1]. Moreover, adult asymptomatic patients have been found to have the same viral loads as symptomatic patients [2]. Studies have shown that age appears to influence whether an infected person is susceptible to illness. Those under the age of 20 years have approximately half the morbidity probability as those over the age of 20 [3]. This improbability of becoming ill from SARS-CoV-2 infection is especially interesting because young children have been found to have 10 to 100 times the viral load as older children and adults, and disproportionately remain asymptomatic [4].

Stem cell growth factor-beta (SCGF-β) has been associated with H7N9 (Asian lineage avian influenza A subtype) and disassociated with H5N1 (a highly pathogenic avian influenza) [5,6]. Elevated SCGF-β has also been associated with specific disease states of hepatocellular cancer, Chagas disease, cardiomyopathy, inflammation and insulin resistance, and unstable carotid plaques [7-10]. Interleukin-16 (IL-16), the second most important variable in predicting SARS-CoV-2 immunity or resistance, has been strongly associated with asthma [11].

Prior studies on the biomarkers associated with SARS-CoV-2 immune response and morbidity include interferon-gamma (IFN-γ), interferon-beta (IFN-β), and interleukin-8 (IL-8) [12]. Other previous research on immune parameters associated with SARS-CoV-2 severity and prognosis have involved interleukin-1 beta (IL-1β) and interleukin-6 (IL-6). However, others found reduced immunoglobin G levels in asymptomatic patients [13,14]. The general finding in prior research regarding the pathogen-immune relationship with SARS-CoV-2 is that symptomatic patients have considerably more inflammation and cytokine storm activity than asymptomatic patients [14].

What has been unknown for SARS-CoV-2 are three questions to which the answers are suggested in this study. First, which immunological variables are statistically significant, and how important is each in predicting asymptomatic status? Second, which of those variables, if any, have a strong negative correlation, or relationship, with disease severity (ie, asymptomatic patients’ levels are significantly higher than symptomatic patients)? And third, is there an algorithmic or formulaic model of prognostic biomarkers that can accurately predict morbidity—who will be asymptomatic if infected, and who is at risk of more severe symptoms and disease progression—and why?

## Methods

This study was based on secondary data published as a supplement in *Nature Medicine* in June 2020 [14]. Therein, immunological factors were measured in 74 patients in the Wanzhou District of China. They were diagnosed as SARS-CoV-2 positive by reverse transcriptase-polymerase chain reaction (RT-PCR) in the 14 days before observations were recorded. The median age of the 37 asymptomatic patients was 41 years (range 8-75 years); 22 were female and 15 were male. For comparison, 37 RT-PCR test–positive patients were selected and matched to the asymptomatic group by age, comorbidities, and sex [14].

In this study, five algorithms, or types, of machine learning—a kind of artificial intelligence employing robust brute-force statistical calculations—were applied to a data set of 74 observations of 34 immunological factors in order to attempt three things: (1) to develop a model to accurately predict which patients will be asymptomatic or symptomatic if infected with SARS-CoV-2; (2) to determine the relative importance of each immunological factor; and (3) to determine if there is any level of a subset of immunological factors that can accurately predict which patients are likely to be immune or resistant to SARS-CoV-2.

Minitab 19, version 19.2020.1 (Minitab LLC), was used to calculate means, 95% CIs, *P* values, and two-sample *t* tests of statistical significance. Correlation coefficients were also computed using Minitab via Spearman rho since the data were distributed nonparametrically. A second classification and regression tree (CART) algorithm was also applied in Minitab to cross-validate decision tree results from R in Rattle. Minitab’s CART methodology was initially described by Stanford University and University of California Berkeley researchers in 1984 [15].

The Rattle library, version 5.3.0 (Togaware), in the statistical programming language R, version 3.6.3 (CRAN), was used to apply five machine learning algorithms—a decision tree, extreme gradient boosting (XGBoost), linear logistic model (LLM), random forest, and support vector machine (SVM)—to learn which model, if any, could predict asymptomatic status and how accurately. Rattle randomly partitioned the data to select and train on 80% (n=59), validate on 10% (n=7), and test on 10% (n=7) of observations. Two evaluation methods were used: (1) plots of linear fits of the predicted versus observed categorization; and (2) a pseudo-R^2^ measure calculated as the square root of the correlation between the predicted and observed values. Pseudo-R^2^ measure results were evaluated twice, each using for evaluation data that were held back by being randomly selected during partitioning and averaging the two accuracy findings for the final results.

Rattle’s rpart decision tree was also used to identify if any levels of one or more immunological factors could accurately diagnose someone as asymptomatic (ie, via rules). The decision tree results reported here used 20 and 12 as the minimum number of observations necessary in nodes before the split (ie, minimum split). The trees used 7 and 4 as the minimum number of observations in a leaf node (ie, minimum bucket).

The random forest analysis in Rattle began by running a series of differently sized random forest algorithms, ranging from 50 to 500 decision trees, to learn the optimum number of trees to minimize error. Each random forest consisted of a minimum of six variables, which was closest to the square root of the number of statistically significant variables (ie, 34). The lowest error rate was approximately 200 decision trees.

The five machine learning models and CART classification trees were run, including and excluding SCGF-β to identify if there were alternative prognostic biomarkers and levels in the immune profile that could accurately classify and predict SARS-CoV-2 immunity.

## Results

In total, 34 of the 53 immunological factors (64.2%) were indicated as statistically significant by *P* values <.05 from a Spearman rho correlation. Of those 34 factors, 31 were statistically significant with *P* values <.01. Conversely, 35.9% of the 53 immune factors had no statistically significant association with whether a patient was asymptomatic or symptomatic to SARS-CoV-2.

The 22 factors positively correlated with being symptomatic ranged from a minimum coefficient of 0.205 (monocyte chemotactic protein-3 [MCP-3]) to a maximum of 0.781 (tumor necrosis factor–related apoptosis-inducing ligand [TRAIL]). The 11 factors negatively associated with being symptomatic ranged from a minimum of –.866 (SCGF-β) to a maximum of –0.276 (interferon alpha-2 [IFNα2]) (see Table 1).

**Table 1:** Immunological factors associated with SARS-CoV-2 morbidity ranked by Spearman correlation coefficients with 95% CIs and *P* values.

Immunological factor	Abbreviation	Pairwise Spearman correlation to asymptomatic (0) or symptomatic (1) status	95% CI	*P* value (<.05 target)
TNF^a^-related apoptosis-inducing ligand	TRAIL	0.781	0.654 to 0.865	<.001
Growth-related oncogene alpha	GRO-α	0.750	0.611 to 0.845	<.001
Macrophage-colony stimulating factor	M-CSF	0.748	0.608 to 0.843	<.001
Interleukin-6	IL-6	0.705	0.549 to 0.813	<.001
Granulocyte-colony-stimulating factor	G-CSF	0.697	0.539 to 0.808	<.001
Interleukin-2	IL-2	0.667	0.499 to 0.787	<.001
Nerve growth factor beta	NGF-β	0.651	0.479 to 0.775	<.001
Interleukin-10	IL-10	0.614	0.431 to 0.748	<.001
Monocyte chemoattractant protein-1	MCP-1	0.594	0.407 to 0.733	<.001
Stem-cell factor	SCF	0.586	0.397 to 0.728	<.001
Interleukin-15	IL-15	0.527	0.325 to 0.683	<.001
Interleukin-8	IL-8	0.514	0.311 to 0.673	<.001
Interferon-gamma	IFN-γ	0.464	0.252 to 0.633	<.001
Interleukin-7	IL-7	0.454	0.240 to 0.625	<.001
Interferon gamma inducible protein-10	INF-γ-IP-10	0.451	0.237 to 0.623	<.001
Interleukin-18	IL-18	0.438	0.223 to 0.613	<.001
Platelet-derived growth factor BB	PDGF-BB	0.436	0.220 to 0.611	<.001
Interleukin-2 receptor alpha	IL-2Rα	0.388	0.166 to 0.572	.001
Immunoglobin G (convalescing)	IgG Conv	0.366	0.143 to 0.544	.001
Monokine-induced by gamma	MIG	0.364	0.140 to 0.552	.001
Immunoglobulin G (acute)	IgG Acute	0.330	0.103 to 0.524	.004
Macrophage migration inhibitory factor	MIF	0.237	0.006 to 0.444	.04
Monocyte chemotactic protein-3	MCP-3	0.205	–0.270 to 0.416	.08^b^
Vascular endothelial growth factor	VEGF	0.184	–0.048 to 0.397	.12^b^
N gene	N	0.180	–0.053 to 0.394	.13^b^
Interleukin-3	IL-3	0.163	–0.070 to 0.379	.17^b^
Interleukin-12-p40	IL-12(p40)	0.151	­–0.082 to 0.368	.20^b^
Interleukin-9	IL-9	0.149	–0.084 to 0.366	.21^b^
Interleukin-1 beta	IL-1β	0.125	­–0.107 to 0.345	.29^b^
Days shed virions	Days shed	0.122	–0.110 to 0.342	.30^b^
Stromal cell-derived factor-1 alpha	SDF-1α	0.098	–0.124 to 0.320	.41^b^
Interleukin-12-p70	IL-12(p70)	0.083	–0.149 to 0.306	.48^b^
Interleukin-17	IL-17	0.067	–0.164 to 0.291	.57^b^
Interleukin-4	IL-4	0.020	–0.210 to 0.247	.87^b^
Interleukin-13	IL-13	–0.022	–0.249 to 0.208	.86^b^
Fibroblast growth factor	FGF	–0.078	–0.302 to 0.153	.51^b^
Regulated upon activation, normal T-cell expressed and secreted	RANTES	–0.085	–0.308 to 0.146	.47^b^
Macrophage inflammatory protein-1 beta	MIP-1β	–0.109	–0.330 to 0.123	.35^b^
ORF1ab gene	ORF1ab	–0.113	–0.334 to 0.119	.34^b^
Macrophage inflammatory protein-1 alpha	MIP-1α	–0.138	–0.356 to 0.095	.24^b^
Tumor necrosis factor-alpha	TNF-α	–0.168	–0.383 to 0.065	.15^b^
Tumor necrosis factor-beta	TNF-β	–0.197	–0.409 to 0.035	.09^b^
Interferon alpha-2	IFNα2	–0.276	–0.478 to –0.046	.02^c^
Leukemia inhibitory factor	LIF	–0.312	–0.509 to –0.840	.007^c^
Interleukin-5	IL-5	–0.316	–0.512 to –0.089	.006^c^
Interleukin-1 alpha	IL-1α	–0.332	–0.526 to –0.106	.004^c^
Granulocyte-macrophage colony-stimulating factor	GM-CSF	–0.359	–0.548 to –0.134	.002^c^
Interleukin-1 receptor alpha	IL-1Rα	–0.359	–0.548 to –0.135	.002^c^
Eotaxin	Eotaxin	–0.390	–0.576 to –0.169	.001^c^
Cutaneous T-cell–attracting chemokine	CTACK	–0.456	–0.627 to –0.243	<.001^c^
Hepatocyte growth factor	HGF	–0.594	–0.733 to –0.407	<.001^c^
Interleukin-16	IL-16	–0.827	–0.895 to –0.721	<.001^c^
Stem-cell growth factor-beta	SCGF-β	–0.866	–0.920 to –0.780	<.001^c^

^a^TNF: tumor necrosis factor.

^b^Statistically insignificant.

^c^Statistically significant negative correlations.

When SCGF-β was included in the machine learning analysis, two algorithms predicted and classified SARS-CoV-2 immunity or resistance by being asymptomatic with 100% accuracy: a decision tree and XGBoost. When SCGF-β was excluded, a random-forest algorithm predicted and classified SARS-CoV-2 asymptomatic and symptomatic cases with 94.8% AUROC (area under the receiver operating characteristic) curve accuracy (95% CI 90.17%-100%) (see Table 2).

**Figure 1:**
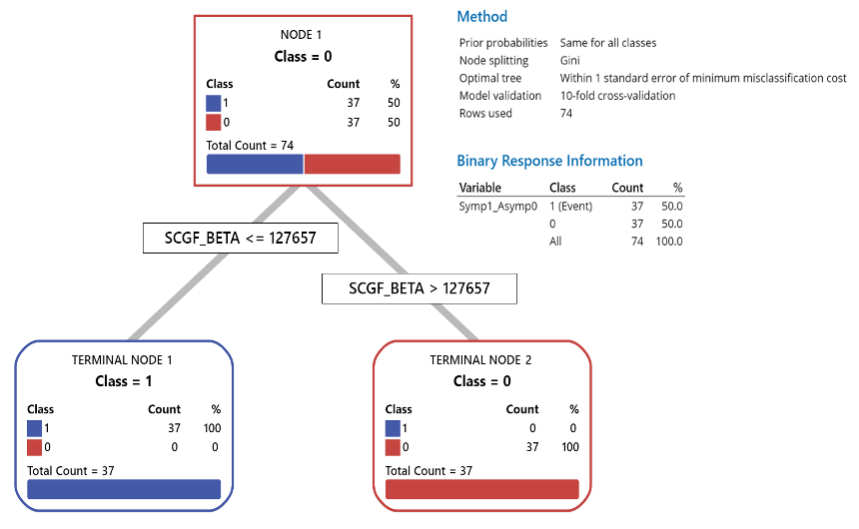
Classification and regression tree (CART) of the role of stem cell growth factor-beta (SCGF-β) in predicting SARS-CoV-2 morbidity.

**Figure 2:**
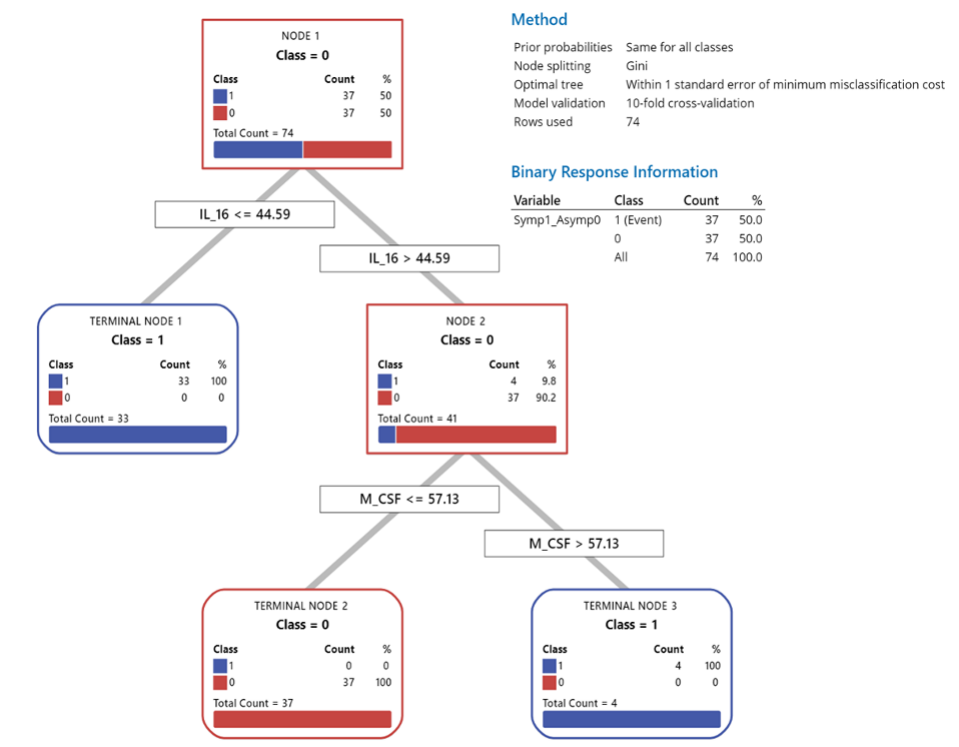
Classification and regression tree (CART) of the role of interleukin-16 (IL-16) and macrophage colony-stimulating factor (M-CSF) in predicting SARS-CoV-2 morbidity in the absence of stem cell growth factor-beta (SCGF-β).

Notably, both the rpart decision trees and CART classification trees independently identified three prognostic biomarkers at specific levels that could classify asymptomatic and symptomatic cases with 95%-100% accuracy. When SCGF- β was included, all asymptomatic cases had levels >127,656.8, while all symptomatic cases had levels <127,656.8 (Figure 1). When SCGF-β was excluded, as a type of contingency analysis to understand prognostic biomarker levels in other factors better, IL-16 accurately classified asymptomatic cases >44.59 and symptomatic cases <44.59 in 90.4% of the cases. In the remaining 9.6% of cases where IL-16 >44.59, all had macrophage colony-stimulating factor (M-CSF) >57.13 (Figure 2).

**Table 2:** Comparative accuracy of six machine learning algorithms in predicting SARS-CoV-2 asymptomatic status from immunological factors.

Machine learning model		Pseudo-R^2^ (10% evaluation holdback sample 1) (%)	Pseudo-R^2^ (10% evaluation holdback sample 2) (%)	Average Pseudo-R^2^ (%)
**With SCGF-β** ^a^				
	Decision tree	100.00	100.00	100.00
	XGBoost^b^	100.00	100.00	100.00
	GLM^c^ (logistic)	100.00	98.89	99.45
	Random forest	99.46	94.83	97.15
	SVM^d^	78.81	96.99	87.90
**Without SCGF-β**				
	Random forest	97.68	91.91	94.80
	GLM (logistic)	100.00	85.96	92.98
	SVM	77.76	89.69	83.73
	XGBoost	99.42	54.27	76.85
	Decision tree	100.00	2.22	51.11

^a^SCGF-β: stem cell growth factor-beta.

^b^XGBoost: extreme gradient boosting.

^c^GLM: generalized linear model.

^d^SVM: support vector machine.

**Figure 3:**
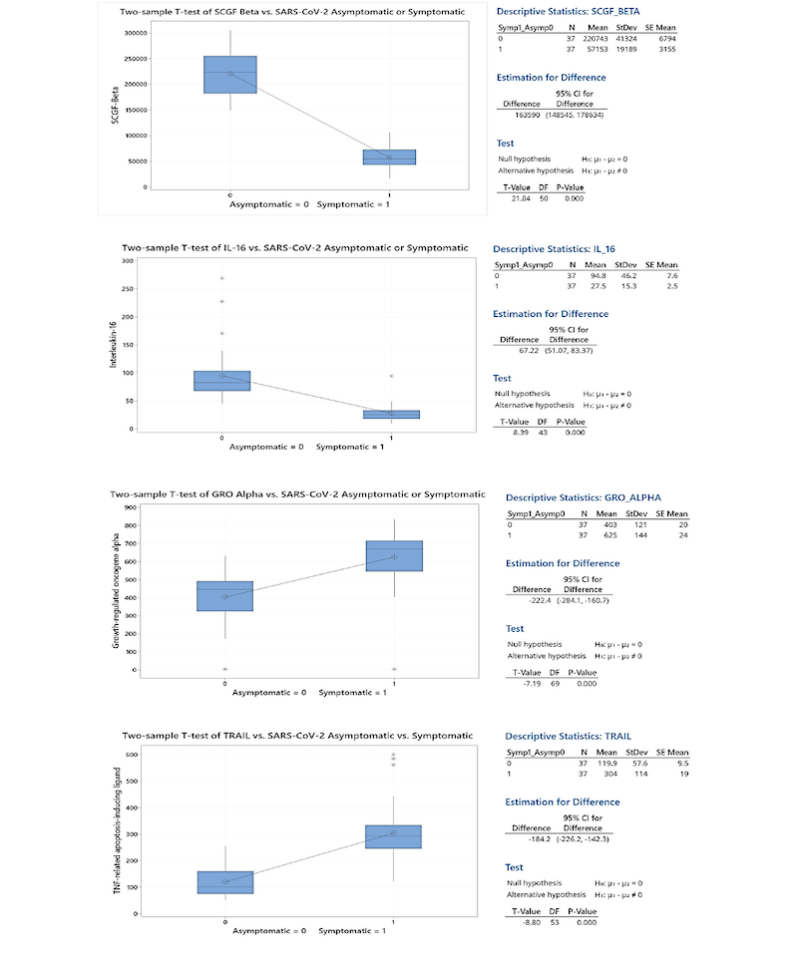
Two-sample t tests of statistical significance of the difference in means of four leading prognostic biomarkers for asymptomatic or symptomatic SARS-CoV-2. SCGF-β: stem cell growth factor-beta; IL-16: interleukin-16; GRO-α: growth-related oncogene alpha; TRAIL: tumor necrosis factor–related apoptosis-inducing ligand.

Two-sample *t* tests for the four factors with the highest positive and negative correlation coefficients, interquartile ranges, outliers, and levels between asymptomatic and symptomatic patients that were statistically significant were computed to ordinally rank factors by their correlation coefficients (Figure 3).

**Figure 4:**
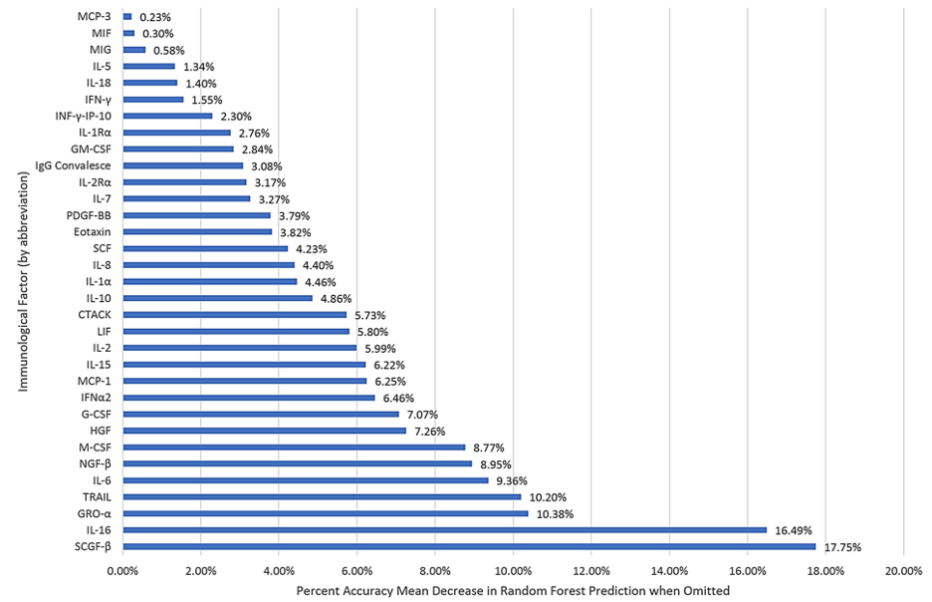
Relative importance of immunological variables from random forest analysis in predicting SARS-CoV-2 morbidity.

A random forest analysis of the most important variables to accurately classify and predict SARS-CoV-2 patients by binary morbidity ordinally ranked the 34 statistically significant factors. Unsurprisingly, SCGF-β, and IL-16, followed by growth-related oncogene alpha (GRO-α) and TRAIL, respectively, were the most critical factors in predicting morbidity (Figure 4).

Finally, the results suggest that IL-1β, 3, 4, 9, 12, 13, 17, and RANTES (regulated upon activation, normal T-cell expressed and secreted) are of low importance, or comparative irrelevance, in the pathogen-immune relationship and, that SCGF-β, IL-16, HGF, INFNα2, LIF, CTACK, IL-1α, Eotaxin, GM-CSF, IL-1Rα, and IL-5 are valuable in models to predict and classify asymptomatic or symptomatic SARS-CoV-2 cases accurately.

## Discussion

### Principal Findings

While it has been speculated that stem cells may play a role in SARS-CoV-2 and other zoonoses’ resistance, prior research has focused on different stem cell involvement than SCGF-β [16-18]. Previous research has also established that stem cells can inhibit viral growth by expressing IFN-γ–stimulated genes and have been particularly effective against influenza A H5N1 virus and resulting lung injuries [19,20]. Stem cell therapy has been hypothesized as a treatment for SARS-CoV-2; however, there is no record in the literature specific as to which factors may influence SARS-CoV-2 infections, favorably or unfavorably, or to what degree until now [21].

Researchers have recently found that symptomatic patients generally have a more robust immune response to SARS-CoV-2 infection, culminating in cytokine storms in the worst cases. Conversely, asymptomatic patients have been found to have a weaker immune response [14]. Because infections are causal to immune response, of particular interest in this study were the most impactful immune-related variables that negatively correlated with asymptomatic status (ie, variables that were greater for asymptomatic patients than symptomatic patients) (marked with a superscripted “c” in Table 1). 

This paper’s overarching importance is the identification of immunological factors for diagnoses, treatments, and preclinical prophylactic immune-based approaches to SARS-CoV-2 in the first 7 months of a pandemic that experts now opine will last decades [22]. Immunostimulant approaches are especially valuable because, unlike antivirals and vaccines, they may be given later in the course of the disease to optimize outcomes [21].

The primary importance of this work is machine learning algorithmic models that can predict with high accuracy whether someone, once infected, will be asymptomatic or symptomatic from SARS-CoV-2. This knowledge gives clinicians new tools to identify populations in advance who appear to be at higher risk of danger from the virus. Such devices, especially once reproduced in a more extensive study, may also inform policy decisions as to who needs to shelter in place. Finally, because of the scale of this pandemic and practical constraints as to how many vaccination doses can be manufactured and how quickly this can be done, such tools may become valuable in prioritizing vaccine administration to those in greatest need because they have a higher biological and immunological risk.

This work’s secondary importance is a description of the cytokine and chemokine profile that is associated with asymptomatic or symptomatic SARS-CoV-2 infections. It enables a better understanding of the pathogen-immune relationship. These profiles provide insights into the biological pathways critical for SARS-CoV-2 progression.

As one example, stem cell factors secrete multiple factors that regulate immune cells and modulate them to restore tissue homeostasis. These results suggest that higher levels of SCF-β (stem-cell factor-beta) may better control immune responses to prevent the more robust reactions universally associated so far with highly symptomatic patients and, further, prevent high morbidity and mortality cytokine storms. A better understanding of the pathogen-immune relationship may enable researchers to prevent and treat patients with SARS-CoV-2 infection more effectively with therapeutics currently untested and unused. This knowledge may also extend to similar zoonotic coronaviruses in the future.

The tertiary importance of this work is identifying three immune factors and precise levels that appear to be prognostic biomarkers as to whether someone, once infected with SARS-CoV-2, will be immune or resistant, as demonstrated by being asymptomatic or not. These insights also suggest new candidates for therapeutic research focused on the relatively newly identified and ill-understood SCGF-β and its role in the immunological process.

The quaternary importance of this work is further proof that machine learning methods can accurately and quickly identify critical elements of disease dynamics that accelerate understanding and improve outcomes during pandemics. Moreover, it is an example of how a “dry” data science laboratory can link to clinical or “wet” laboratory science for real-world applications.

### Limitations

This study has several limitations. First, it is unknown from the data set how many days passed between exposure to the virus and immunological testing, or whether it was universally the same number of days. Second, because immune profiles are temporally sensitive, ideally, several tests would have been taken over several days, which did not occur (R Jankord, PhD, July 22, 2020). Third, immunological signaling and processing are multifactorial and complex. Therefore, it is unclear why SCGF-β levels are categorically high in asymptomatic patients and low in symptomatic patients, or whether they are causal to SARS-CoV-2 response. Fourth, combinatorial and sequential analysis of these immunological elements may be an important future research area to optimize therapeutic research outcomes. Fifth, at least one study in a leading journal, *The Lancet*, found that Chinese SARS-CoV-2 case data may have been misreported by as much as 400% [23]. That study, and much higher case and fatality numbers in over 200 countries, have created distrust and skepticism of SARS-CoV-2–related data originating from China.

Future research could ameliorate these limitations and focus on a more extensive study group to attempt to reproduce the results. Moreover, a prospective case-control study of patients with decreased SCGF-β levels and supplementation that was protective against SARS-CoV-2 severity and symptoms would be invaluable validation.

### Conclusion

One implication of these findings is that if we can predict the 80% of society who may be immune or resistant to SARS-CoV-2, or asymptomatic, it may profoundly impact public health intervention decisions as to who needs to be protected and by how much. If, for example, 80% of the shelter-in-place orders and the resultant dramatic reduction in economic and social activity could have been prevented by accurately predicting who is at low risk of infection, the economic benefits alone may have been valued in US$ trillions. The second implication of these findings is evidence that elevated levels of SCGF-β, IL-16, and M-CSF may have a causal relationship with SARS-CoV-2 immunity or resistance, and may have utility as diagnostic determinants to (1) inform public health policy decisions to prioritize and reduce shelter-in-place orders to minimize economic and social impacts; (2) advance therapeutic research; and (3) prioritize vaccine distribution to benefit those with the greatest need and risks first.

## Abbreviations

AUROC: area under the receiver operating characteristic
GRO-α: growth-related oncogene alpha
IFN-β: interferon-beta
IFN-γ: interferon-gamma
IFNα2: interferon alpha-2
IL-1β: interleukin-1 beta
IL-6: interleukin-6
IL-8: interleukin-8
IL-16: interleukin-16
LLM: linear logistic model
M-CSF: macrophage colony-stimulating factor
MCP-3: monocyte chemotactic protein-3
RANTES: regulated upon activation, normal T-cell expressed and secreted
RT-PCR: reverse transcriptase-polymerase chain reaction
SCF-β: stem-cell factor-beta
SCGF-β: stem cell growth factor-beta
SVM: support vector machine
TRAIL: tumor necrosis factor–related apoptosis-inducing ligand
XGBoost: extreme gradient boosting
